# Supplementary material for: The Influence of Gender in The Prognostic Impact of Diabetes mellitus in acute Pulmonary Embolism
Source: J Clin Med. 2020 Oct 30;9(11):3511. doi: 10.3390/jcm9113511 (PMC7693483; doi:10.3390/jcm9113511)
Supplement: Supplementary file 1 [file jcm-09-03511-s001.pdf]

Table S1. Comparison between survivors and non-survivors

**Characteristic**	**Survivors (n=337)**	**Non-survivors (240)**	**p-value**
**Male gender, n (%)**	113 (33.5)	100 (51.7)	0.05
**Age (years), mean (SD)**	59 (18)	75 (14)	<0.001
**Diabetes *mellitus*, n (%)**	49 (14.5)	65 (27.1)	<0.001
**Arterial Hypertension, n (%)**	170 (50.4)	148 (61.7)	0.08
**Haemoglobin (g/dL), mean (SD)**	12.6 (2.2)	12.3 (2.3)	0.15
**Creatinine (mg/dL), median (IQR)**	0.84 (0.70-1.10)	1.13 (0.87-1.66)	<0.001
**Lymphocytes (cells/μL), median (IQR)**	1780 (1200-2350)	1320 (848-1975)	<0.001
**Platelets**	213 (167-274)	190 (152-259)	0.01
**C-Reactive Protein (mg/L), median (IQR)**	38.7 (14.2-85.5)	42.0 (18.5-102.1)	0.13
**BNP (pg/mL), median (IQR)**	180.1 (49.1-450.5)	438.2 (173.5-1024.3)	<0.001
**Central/bilateral PE, n (%)**	115 (34.2)	60 (25.1)	0.02
**Thrombolytic therapy, n (%)**	44 (13.1)	23 (9.6)	0.20

BNP: B type natriuretic peptide; IQR: interquartile range; PE: pulmonary embolism; SD: standard deviation
